# Supplementary material for: From sequence to enzyme mechanism using multi-label machine learning
Source: BMC Bioinformatics. 2014 May 19;15:150. doi: 10.1186/1471-2105-15-150 (PMC4229970; doi:10.1186/1471-2105-15-150)
Supplement: Additional file 2 — Java code of ml2db. Additional file ml2db_code.tar.gz contains the Java source code to run the multi-label machine learning experiments and save the results to database. The code’s Javadoc is included. [file 1471-2105-15-150-S2.zip › additional file 2/ml2db/utils/doc/uk/ac/ed/inf/utils/database/DbUtils.html]

DbUtils


---


|  |  |  |
| --- | --- | --- |
| **Overview** \| **Package** \| **Class** \| **Use** \| **Tree** \| **Deprecated** \| **Index** \| **Help** | |  |
| **PREV CLASS**   **NEXT CLASS** | **FRAMES**    **NO FRAMES**     **All Classes** |  |
| SUMMARY: NESTED \| FIELD \| CONSTR \| METHOD | DETAIL: FIELD \| CONSTR \| METHOD |  |


---


## uk.ac.ed.inf.utils.database Class DbUtils

```
java.lang.Object
  uk.ac.ed.inf.utils.database.DbUtils
```

---

``` public class DbUtils extends java.lang.Object ```

Database utilities (to create sql code etc.)

**Version:**
:   20061125

**Author:**
:   Luna

---

| **Field Summary** | |
| --- | --- |
| `static java.lang.String` | `AUTO_INCREMENT_SQL_DATATYPE` |
| `static java.lang.String` | `BOOLEAN_SQL_DATATYPE` |
| `static java.lang.String` | `CHAR_2_SQL_DATATYPE` |
| `static java.lang.String` | `CHAR_4_SQL_DATATYPE` |
| `static java.lang.String` | `DOUBLE_QUOTE_CHARACTER` |
| `static java.lang.String` | `DOUBLE_SQL_DATATYPE` |
| `static java.lang.String` | `END_TIMESTAMP` |
| `static java.lang.String` | `ID` |
| `static java.lang.String` | `INTEGER_SQL_DATATYPE` |
| `static java.lang.String` | `REVERSED_PRIME_CHARACTER` |
| `static java.lang.String` | `SINGLE_QUOTE_CHARACTER` |
| `static java.lang.String` | `START_TIMESTAMP` |
| `static java.lang.String` | `TEXT_SQL_DATATYPE` |
| `static java.lang.String` | `TIMESTAMP` |
| `static java.lang.String` | `TIMESTAMP_DOWNLOADED` |
| `static java.lang.String` | `TIMESTAMP_SQL_DATATYPE` |
| `static java.lang.String` | `VARCHAR_90_SQL_DATATYPE` |


**Constructor Summary**

`DbUtils()`


| **Method Summary** | |
| --- | --- |
| `static boolean` | `elementExistsInTable(java.lang.String table, java.lang.String column, java.lang.String element, DbConn dbConn)`             Get if an element exists in a table column |
| `static boolean` | `elementsExistsInTable(java.lang.String table, java.lang.String column1, java.lang.String element1, java.lang.String column2, java.lang.String element2, DbConn dbConn)`             Get if two elements exists in the same table row |
| `static boolean` | `existsInTable(java.lang.String query, DbConn dbConn)` |
| `static int` | `getBasicTypeOfDataColumn(java.util.ArrayList<java.lang.String> list)`             Given a list of strings, it returns a generic data type for the database column that could best contain them: INTEGER, DOUBLE, STRING If the strings passes a parseInteger: type = INTEGER if the strings contain digits, 'E' (for exponential), dot, plus, minus: type = DOUBLE If the strings contain letters or other characters: type = STRING Note: If the string contains only digits, but also intervening white spaces: type = STRING |
| `static java.util.ArrayList<java.util.ArrayList<java.lang.String>>` | `getColumnsfrom2DArray(java.lang.String[][] array)`             Takes a 2 dimensional array of data and returns the columns (as an array list of array lists) |
| `static java.lang.String` | `getDataTypeForAnArray(java.util.ArrayList<java.lang.String> list)`             Guesses from data type and length the appropriate SQL type of a column to contain the data in the array |
| `static TableColumn` | `getIdColumn()`             get the definition for an "id" column of type auto-increment (which is primary key) |
| `static java.lang.String` | `getListOfValuesString(java.util.Vector<java.lang.String> listOfValues, java.lang.String quotationCharacter)`             Transforms a list of values into a comma separated list |
| `static java.sql.ResultSet` | `getResultSet(java.lang.String sql, DbConn dbConn)`             Executes an sql query returning a result set |
| `static java.util.Vector<java.lang.String>` | `getResultSetColumnAsVector(java.sql.ResultSet rs, int column)`             Takes a resultSet and returns an array of Strings |
| `static TableColumn` | `getTimeDownloadedColumn()` |
| `static TableColumn` | `getTimestampColumn()` |
| `static boolean` | `insert(DbConn conn, java.lang.String insertSql)`             Executes sql insert and returns whether the insert was successful. |
| `static java.lang.Integer` | `insertReturnsKey(DbConn conn, java.lang.String insertSql)`             Executes sql insert and returns the auto generated key for the first inserted row. |
| `static boolean` | `isValidConnection(java.sql.Connection conn)`             Test Validity of JDBC Installation. |
| `static void` | `main(java.lang.String[] args)`             Runs the 'analyze table ' command for all the tables in a mysql database |
| `static void` | `printResultsSet(java.sql.ResultSet results)`             Prints the results of a query |
| `static java.lang.String` | `resultsSetToString(java.sql.ResultSet results)`             Generates a string for the results of a query |
| `static boolean` | `similarElementExistsInTable(java.lang.String table, java.lang.String column, java.lang.String element, DbConn dbConn)` |
| `static boolean` | `testConnection(java.sql.Connection conn, java.lang.String query)`             Test Validity of a Connection. |

**Methods inherited from class java.lang.Object**

`equals, getClass, hashCode, notify, notifyAll, toString, wait, wait, wait`

**Field Detail**

### AUTO\_INCREMENT\_SQL\_DATATYPE

```
public static final java.lang.String AUTO_INCREMENT_SQL_DATATYPE
```

**See Also:**: Constant Field Values

---


### BOOLEAN\_SQL\_DATATYPE

```
public static final java.lang.String BOOLEAN_SQL_DATATYPE
```

**See Also:**: Constant Field Values

---


### CHAR\_2\_SQL\_DATATYPE

```
public static final java.lang.String CHAR_2_SQL_DATATYPE
```

**See Also:**: Constant Field Values

---


### CHAR\_4\_SQL\_DATATYPE

```
public static final java.lang.String CHAR_4_SQL_DATATYPE
```

**See Also:**: Constant Field Values

---


### DOUBLE\_SQL\_DATATYPE

```
public static final java.lang.String DOUBLE_SQL_DATATYPE
```

**See Also:**: Constant Field Values

---


### END\_TIMESTAMP

```
public static final java.lang.String END_TIMESTAMP
```

**See Also:**: Constant Field Values

---


### ID

```
public static final java.lang.String ID
```

**See Also:**: Constant Field Values

---


### TIMESTAMP

```
public static final java.lang.String TIMESTAMP
```

**See Also:**: Constant Field Values

---


### INTEGER\_SQL\_DATATYPE

```
public static final java.lang.String INTEGER_SQL_DATATYPE
```

**See Also:**: Constant Field Values

---


### START\_TIMESTAMP

```
public static final java.lang.String START_TIMESTAMP
```

**See Also:**: Constant Field Values

---


### TIMESTAMP\_DOWNLOADED

```
public static final java.lang.String TIMESTAMP_DOWNLOADED
```

**See Also:**: Constant Field Values

---


### TIMESTAMP\_SQL\_DATATYPE

```
public static final java.lang.String TIMESTAMP_SQL_DATATYPE
```

**See Also:**: Constant Field Values

---


### VARCHAR\_90\_SQL\_DATATYPE

```
public static final java.lang.String VARCHAR_90_SQL_DATATYPE
```

**See Also:**: Constant Field Values

---


### TEXT\_SQL\_DATATYPE

```
public static final java.lang.String TEXT_SQL_DATATYPE
```

**See Also:**: Constant Field Values

---


### DOUBLE\_QUOTE\_CHARACTER

```
public static final java.lang.String DOUBLE_QUOTE_CHARACTER
```

**See Also:**: Constant Field Values

---


### SINGLE\_QUOTE\_CHARACTER

```
public static final java.lang.String SINGLE_QUOTE_CHARACTER
```

**See Also:**: Constant Field Values

---


### REVERSED\_PRIME\_CHARACTER

```
public static final java.lang.String REVERSED_PRIME_CHARACTER
```

**See Also:**: Constant Field Values


**Constructor Detail**

### DbUtils

```
public DbUtils()
```


**Method Detail**

### elementExistsInTable

```
public static boolean elementExistsInTable(java.lang.String table,
                                           java.lang.String column,
                                           java.lang.String element,
                                           DbConn dbConn)
```

:   Get if an element exists in a table column

    :   **Parameters:**: `table` - table name: `column` - column name: `element` - the desired string: `dbConn` - the database connection **Returns:**: true if the element exists in the database table

---


### elementsExistsInTable

```
public static boolean elementsExistsInTable(java.lang.String table,
                                            java.lang.String column1,
                                            java.lang.String element1,
                                            java.lang.String column2,
                                            java.lang.String element2,
                                            DbConn dbConn)
```

:   Get if two elements exists in the same table row

    :   **Parameters:**: `table` - table name: `column1` - column1 name: `element1` - the desired string in column 1: `column2` - column2 name: `element2` - the desired string in column 2: `dbConn` - the database connection **Returns:**: true if the element exists in the database table

---


### existsInTable

```
public static boolean existsInTable(java.lang.String query,
                                    DbConn dbConn)
```

---


### getBasicTypeOfDataColumn

```
public static int getBasicTypeOfDataColumn(java.util.ArrayList<java.lang.String> list)
```

:   Given a list of strings, it returns a generic data type for the database
    column that could best contain them: INTEGER, DOUBLE, STRING
    If the strings passes a parseInteger: type = INTEGER if the strings
    contain digits, 'E' (for exponential), dot, plus, minus: type = DOUBLE If
    the strings contain letters or other characters: type = STRING Note: If
    the string contains only digits, but also intervening white spaces: type
    = STRING

    :   **Parameters:**: `list` - a list of strings (data) **Returns:**: the basic datatype (INTEGER, DOUBLE, STRING)

---


### getColumnsfrom2DArray

```
public static java.util.ArrayList<java.util.ArrayList<java.lang.String>> getColumnsfrom2DArray(java.lang.String[][] array)
```

:   Takes a 2 dimensional array of data and returns the columns (as an array
    list of array lists)

    :   **Parameters:**: `array` - a 2 dimentional array containing data **Returns:**: a list containing one array list per column of data

---


### getDataTypeForAnArray

```
public static java.lang.String getDataTypeForAnArray(java.util.ArrayList<java.lang.String> list)
```

:   Guesses from data type and length the appropriate SQL type of a column to
    contain the data in the array

    :   **Parameters:**: `list` - an array of strings **Returns:**: the SQL type of a column for the data in the array

---


### getIdColumn

```
public static TableColumn getIdColumn()
```

:   get the definition for an "id" column of type auto-increment (which is
    primary key)

---


### getListOfValuesString

```
public static java.lang.String getListOfValuesString(java.util.Vector<java.lang.String> listOfValues,
                                                     java.lang.String quotationCharacter)
```

:   Transforms a list of values into a comma separated list

    :   **Parameters:**: `listOfValues` - e.g. [a, b, c]: `the` - quotation character to surround the values (usually double quotes or apex) **Returns:**: a comma separated list, for example (if the quotation character is an apex): 'a', 'b', 'c'

---


### getResultSet

```
public static java.sql.ResultSet getResultSet(java.lang.String sql,
                                              DbConn dbConn)
```

:   Executes an sql query returning a result set

    :   **Parameters:**: `sql` - an sql query **Returns:**: a resultSet **Throws:**: `java.sql.SQLException`

---


### getResultSetColumnAsVector

```
public static java.util.Vector<java.lang.String> getResultSetColumnAsVector(java.sql.ResultSet rs,
                                                                            int column)
```

:   Takes a resultSet and returns an array of Strings

    :   **Parameters:**: `rs` - a ResultSet: `column` - the column number (1 = the first column) **Returns:**: a `Vector` with the query results

---


### getTimeDownloadedColumn

```
public static TableColumn getTimeDownloadedColumn()
```

---


### getTimestampColumn

```
public static TableColumn getTimestampColumn()
```

---


### insert

```
public static boolean insert(DbConn conn,
                             java.lang.String insertSql)
```

:   Executes sql insert and returns whether the insert was successful.

    :   **Parameters:**: `conn` -: `insertSql` - **Returns:**: true if the insert was successful

---


### insertReturnsKey

```
public static java.lang.Integer insertReturnsKey(DbConn conn,
                                                 java.lang.String insertSql)
```

:   Executes sql insert and returns the auto generated key for the first
    inserted row.

    :   **Parameters:**: `conn` -: `insertSql` - **Returns:**: the auto generated key for the first inserted row

---


### isValidConnection

```
public static boolean isValidConnection(java.sql.Connection conn)
```

:   Test Validity of JDBC Installation. Taken from:
    http://www.java2s.com/Code
    /Java/Database-SQL-JDBC/CheckJDBCInstallationforMySQL.htm (author
    unknown)

    :   **Parameters:**: `conn` - a JDBC connection object **Returns:**: true if a given connection object is a valid one; otherwise return false.

---


### main

```
public static void main(java.lang.String[] args)
```

:   Runs the 'analyze table ' command for all the tables in a
    mysql database

    :   **Parameters:**: `args` - the first argument should be the full path to a properties file containing server, user etc. for the database connection **Throws:**: `java.sql.SQLException`

---


### printResultsSet

```
public static void printResultsSet(java.sql.ResultSet results)
```

:   Prints the results of a query

    :   **Parameters:**: `results` - a query results set

---


### resultsSetToString

```
public static java.lang.String resultsSetToString(java.sql.ResultSet results)
```

:   Generates a string for the results of a query

    :   **Parameters:**: `results` - a query results set

---


### similarElementExistsInTable

```
public static boolean similarElementExistsInTable(java.lang.String table,
                                                  java.lang.String column,
                                                  java.lang.String element,
                                                  DbConn dbConn)
```

---


### testConnection

```
public static boolean testConnection(java.sql.Connection conn,
                                     java.lang.String query)
```

:   Test Validity of a Connection. From:
    http://www.java2s.com/Code/Java/Database
    -SQL-JDBC/CheckJDBCInstallationforMySQL.htm

    :   **Parameters:**: `conn` - a JDBC connection object: `query` - a sql query to test against database connection **Returns:**: true if a given connection object is a valid one; otherwise return false.


---


|  |  |  |
| --- | --- | --- |
| **Overview** \| **Package** \| **Class** \| **Use** \| **Tree** \| **Deprecated** \| **Index** \| **Help** | |  |
| **PREV CLASS**   **NEXT CLASS** | **FRAMES**    **NO FRAMES**     **All Classes** |  |
| SUMMARY: NESTED \| FIELD \| CONSTR \| METHOD | DETAIL: FIELD \| CONSTR \| METHOD |  |


---
